# A Survey of Brain Tumor Segmentation and Classification Algorithms

**DOI:** 10.3390/jimaging7090179

**Published:** 2021-09-06

**Authors:** Erena Siyoum Biratu, Friedhelm Schwenker, Yehualashet Megersa Ayano, Taye Girma Debelee

**Affiliations:** 1 College of Electrical and Mechanical Engineering, Addis Ababa Science and Technology University, Addis Ababa 120611, Ethiopia; iranasiyoum@gmail.com (E.S.B.); tayegirma@gmail.com (T.G.D.); 2 Institute of Neural Information Processing, Ulm University, 89081 Ulm, Germany; 3 Ethiopian Artificial Intelligence Center, Addis Ababa 40782, Ethiopia; yehualeuven@gmail.com

**Keywords:** brain tumor, classification, segmentation, region growing, shallow machine learning, deep learning

## Abstract

A brain Magnetic resonance imaging (MRI) scan of a single individual consists of several slices across the 3D anatomical view. Therefore, manual segmentation of brain tumors from magnetic resonance (MR) images is a challenging and time-consuming task. In addition, an automated brain tumor classification from an MRI scan is non-invasive so that it avoids biopsy and make the diagnosis process safer. Since the beginning of this millennia and late nineties, the effort of the research community to come-up with automatic brain tumor segmentation and classification method has been tremendous. As a result, there are ample literature on the area focusing on segmentation using region growing, traditional machine learning and deep learning methods. Similarly, a number of tasks have been performed in the area of brain tumor classification into their respective histological type, and an impressive performance results have been obtained. Considering state of-the-art methods and their performance, the purpose of this paper is to provide a comprehensive survey of three, recently proposed, major brain tumor segmentation and classification model techniques, namely, region growing, shallow machine learning and deep learning. The established works included in this survey also covers technical aspects such as the strengths and weaknesses of different approaches, pre- and post-processing techniques, feature extraction, datasets, and models’ performance evaluation metrics.

## 1. Introduction

Machine learning has been applied in different sectors, the majority of the studies indicate that it was applied in agriculture [1], and health sectors [2,3] for disease detection, prediction, and classifications. In health sectors the most researched areas are breast cancer segmentation and classification [4,5,6,7], brain tumor detection and segmentation [8], and lung and colon cancer segmentation and classification [3].

The gold standard in brain tumor diagnosis is biopsy which includes resection and pathological examination using various cellular (histologic) examination techniques. However, the diagnosis using biopsy is invasive that may result in bleeding and even injury that results in functional loss [9]. As a result, non-invasive brain tumor diagnosis using magnetic resonance imaging is the mainstay of modern neuroimaging that enables physician to characterize structural, cellular, metabolic, and functional properties of brain tumor [9,10].

In a conventional structural MRI scan, a healthy brain contains white mater (WM), gray matter (GM), cerebrospinal fluid (CSF)  [11]. The main variation of these tissues in a structural MRI scan depends on their water content. The white matter (WM), which is 70% water, is a myelinated axon that connects the cerebral cortex with other brain regions. Furthermore, it carries information between neurons and connects the right and left hemispheres of the brain. The gray matter, which is 80% water, contains neuronal and glial cells that control brain activity, and the basal nuclei which are located deep within the white matter. Whereas, the cerebrospinal fluid is almost 100% water, and fills the space between the infoldings of the brain, between the brain and skull, and between the ventricular system in the brain[11,12].

Clinically, due to the variability in size, locality, rate of growth, and pathology, it is difficult to understand the manifestation of a brain tumor. However, a brain tumor is an abnormal mass of tissue, in which some cells grow and multiply uncontrollably. This uncontrollable growth takes up space within the skull and interferes with normal brain activity and damages the brain cells. The damage may be caused through increasing pressure in the brain, by shifting the brain or pushing against the skull, and by invading nerves and healthy brain tissues [13,14]. Different criteria can be used to classify brain tumor. A layered based tumor classification schema that has been proposed by WHO provides a detailed classification techniques that is more pertinent to radiological use. In this schema the hierarchy from top to bottom four layers, that are, final integrated diagnosis, histologic classification, WHO grade, molecular information [15]. However, brain tumors can be more generally grouped into primary and secondary (metastatic) tumors depending on their place of origin [16]. Primary brain tumors originates in the brain itself and are named for the cell types from which they originated. These primary tumors can be benign (non-cancerous) and malignant (cancerous). Benign tumors grow slowly and do not spread elsewhere or invade the surrounding tissues. However, they can put pressure on the brain and compromise its function. On the contrary, the malignant tumors grow rapidly and spread to surrounding tissues. On the other hand, secondary brain tumors originate from another part of the body. These tumors mainly occur due to cancer cells from somewhere else in the patient’s body that spread to the brain. The most common causes of secondary brain tumors are lung cancer, breast cancer, melanoma, kidney cancer, bladder cancer, certain sarcomas, and testicular and germ cell tumors [13,16,17]. Each of these tumors has unique clinical, radiographic, and biological characteristics [13].

In MRI scanning, brain examination can be normal or abnormal. The normal brain tissues in MRI are characterized by gray matter (GM), white matter (WM), and cerebrospinal fluid (CSF) tissues. Apart from the normal tissues listed earlier the tumorous brain scan often contains core tumor, necrosis, and edema. Necrosis is a dead cell located inside a core tumor, while edema is located near active tumor borders. Edema is a swelling that exists due to trapped fluids around a tumor. It can be vasogenic in non-infiltrative extra-axial tumors, such as meningioma, or it can be infiltrative that invades WM tracts of a brain in tumors, such as glioma [10,18]. Furthermore, these tissues often have indistinguishable intensity features in structural MRI sequences, such as T1-w, T2-w, FLAIR. For instance, the difficulty in differentiating between the core tumor and associated inflammation was discussed [19]. In addition to that, Alves et al. [19] demonstrated the difficulty in differentiating tumors using signal intensities alone. They demonstrated using a case where two patients were diagnosed with two different brain tumor types due to both tumors have similar intensity features and both are surrounded by extensive edema.

### 1.1. Brain Tumor Imaging Modalities

There are a variety of imaging techniques used to study brain tumors, such as magnetic resonance imaging (MRI), computed tomography (CT), positron emission tomography (PET), and single-photon emission computed tomography (SPECT) imaging. However, CT and MR imaging are the most widely used techniques, because of their widespread availability and their ability to produce high-resolution images of normal anatomic structures and pathologies [20].

#### 1.1.1. Magnetic Resource Imaging

Magnetic resonance imaging (MRI) of a brain generates several 3-dimensional image data that comprise the three anatomical views of a brain (axial, sagittal, and coronal) at different depths of a brain. Depending on the strength of the magnetic field and the sampling protocols, the image quality, slice thickness, and inter-slice gap vary [21,22]. During MR imaging, a patient lay in a strong magnetic field, almost 10,000 times stronger than the earth’s magnetic field, that forces the protons in the water molecule of the body to align in either a parallel (low energy) or anti-parallel (high energy) orientation with the magnetic field. Then, a radiofrequency pulse is introduced that forces the spinning protons to move out of the equilibrium state. When a radiofrequency pulse pauses, the protons return to an equilibrium state and produce a sinusoidal signal at a frequency dependent on the local magnetic field. Finally, a radio antenna within the scanner detects the sinusoidal signal and creates the image [22,23]. The amount of signal produced by specific tissue types is determined by their number of mobile hydrogen protons, the speed at which they are moving, the time needed for the protons within the tissue to return to their original state of magnetization (T1), and the time required for the protons perturbed into coherent oscillation by the radiofrequency pulse to lose their coherence (T2) relaxation times. As T1 (spin-lattice, also known as longitudinal relaxation) and T2 (spin-spin, also known as traversal relaxation) times are time-dependent, the timing of the radio frequency pulse and the reading of the radiated RF energy change the appearance of the image. In addition, the repetition time (TR) describes the time between successive applications of RF pulse sequences, and the echo time (TE) tells the delay before the RF energy radiated by the tissue in question is measured. The variation of T1 and T2 relaxation times between tissues gives image contrast on T1- and T2-weighted (T1-w and T2-w) images. The T1-w sequence is characterized by short TR and short TE while the T2-w sequence is characterized by long TR and short TE. Tissues with shorter T1 (for example, white matter) appear brighter when compared to tissues with a longer T1 (for example, gray matter) in magnetic resonance images. The other intermediate sequence that adopts long TR from T2-w and short TE from T1-w is a proton density-weighted (PD-w). In PD-w, the number of protons per unit volume in tissues is the main factor in determining the formation of image [23,24].

In the current neuroimaging techniques different MRI brain scan procedures can be performed, these include, the conventional structural MRI, functional MRI, diffusion-weighted imaging (DWI), and diffusion tensor imaging (DTI) [10]. In structural MRI procedure which mainly differentiates healthy and abnormal brain tissues based on their water molecule content is the most commonly employed standard imaging technique. This procedure helps to visualize healthy brain tissues and to map gross brain anatomy, tumoral vascularity, calcification, and radiation-induced micro hemorrhage [10,11]. The structural sequences include T1-w, T2-w, FLAIR, and contrast-enhanced T1-w  [10]. The functional MRI (fMRI) on the other hand is used to capture the neural activity inside a brain through the ratio of oxygenated to the deoxygenated level of blood in the neighboring vasculature while performing a cognitive or motor task. The fMRI is used to localize eloquent cortex and differentiate between tumor grades [10]. The DWI captures the random motion of water molecules in a brain and it is used to characterize a tumor through identification of its cellularity and hypoxia, peritumoral edema, the integrity of WM tracts, and to differentiate between posterior fossa tumors [10,25]. Whereas, diffusion tensor imaging (DTI) is used to analyze the 3D diffusion direction, also known as diffusion tensor, of the water molecule. The DTI helps to determine local effects of the tumor on white matter tract integrity including tract displacement, the existence of vasogenic edema, tumor infiltration, and tract destruction [26].

#### 1.1.2. Computed Tomography Imaging

A computed tomography (CT) scan was used in neuroimaging to help understand the functional and structural status of clinically significant signs of diseases. However, it provides less information than an MRI in brain tumor diagnosis. For instance, CT is inferior to MRI in the characterization of soft tissues like a brain and its use of ionizing radiation. However, a computed tomography (CT) scan can provide more detailed images of the bone structures near a brain tumor, such as the skull or spine. A CT scan may also be used to diagnose a brain tumor if the patient has implants like a pacemaker and when an MRI is not available. Currently, a CT is commonly used in the diagnosis of diseases like acute hemorrhage Parkinson’s, head trauma, and in determining age [27,28]. Therefore, in this survey work, brain tumor segmentation and classification techniques that use the brain scan image of MRI are only explored.

The remaining part of the paper is organized as follows, Section 2 illustrates related works to this survey work and shows their strengths and limitations. In Section 3, the literature search strategy, including the chronological span, journal databases, the keywords used for search, and the inclusion and exclusion criteria, is presented. In Section 4, the commonly used model performance metrics in evaluating the performance of brain tumor segmentation and classification algorithms are highlighted. In Section 5, different region growing, conventional shallow supervised machine learning, and deep learning-based brain tumor segmentation techniques are discussed. Furthermore, the reported performances are presented. The techniques used in conventional machine learning-based brain tumor classification and their classification performance are elaborated in Section 6. In addition, different deep learning models based brain tumor classification techniques with their reported performance are presented. Finally, the paper presents a discussion on Section 7 and a conclusion in Section 8.

## 2. Related Works

The quest to find a better autonomous brain tumor segmentation and classification technique that can aid physicians in brain tumor diagnosis have been an active research area. As a result, several survey works have been completed to foster the research in the field and recap techniques used in brain tumor segmentation and classification. In Table 1, only some of the recent pieces of literature that are related to our survey work are listed. Furthermore, their strengths and limitations are clearly discussed.

Our work is tailored to provide a comprehensive survey of recently proposed different brain tumor segmentation and classification techniques, including region growing, shallow machine learning, and deep learning. The established work in this survey also covers technical aspects, such as the strengths and weaknesses of different approaches, together with their performance.

## 3. Method

In this survey work, peer reviewed research papers from 2015 to 2021 that were published on Scopus and Web of Science indexed journals are surveyed to investigate the region growing, deep learning based brain tumor segmentation techniques, and machine learning and deep learning based brain tumor classification techniques. The databases that are extensively searched for this survey work were: (1) IEEE Xplore Digital Library, (2) Science Direct, (3) PubMed, (4) Google Scholar, and (5) MDPI. The search criterion includes (“Brain Tumor”) AND (“Region Growing”) AND (“Segmentation”) AND (“Deep Learning”) AND ("Machine Learning") AND ("Classification"). The methodology used for selecting literature is clearly shown in Algorithm algorithm1. In addition, the paper inclusion criteria (IC) and exclusion criteria (EC) is indicated on Table 2.
**Algorithm 1** Paper search strategy from different search databases.1:**procedure**Topic(Application of Machine Learning and Region Growing Techniques in Brain Tumor Segmentation and Classification)2:     *SearchDatabases* ←*IEEEX plore, GoogleScholar, ScienceDirect, PubMed, MDPI*3:    *SearchYear*←2015−2021 **AND** *Few papers from older years asexceptional to enrich* Section 14:     *i*← 1                                                 ▹ Initialize counter5:     *N*← 5                                        ▹ N is the number of search databases6: 7:    **for** *i* ≤ *N* **do**8:         *Keyword* ← *braintumor, deeplearning, machinelearning, regiongrowing, segmentation, classi fication*9:        **if**  *SearchLink* ∈*SearchDatabases* **and** *Year* ∈*SearchYear* **then**10:           Search (Brain Tumor **AND** Region Growing **AND** Segmentation **AND** Deep Learning **AND** Machine Learning **AND** Classification)11:        **end if**12:    **end for**13:    **if** *NumberofPapers* ≥ 0 **then**14:        **Refine Papers**15:        *ApplyInclusionCriteria*← *IC*1, *IC*2, *IC*316:        *ApplyExclusionCriteria*← *EC*1, *EC*2, *EC*317:    **end if**18:**end procedure**

## 4. Performance Measuring Metrics

Evaluating the segmentation and classification performance of a machine learning algorithm is an essential part of a research project. A machine learning model may give a satisfying result when evaluated using a metric, for instance, accuracy score but may give poor results when evaluated against other metrics such as precision or any other metric. Therefore, most of the time various evaluation metrics are applied to measure and compare the model performance.

In a segmentation task, true positive (TP) represents a pixel that is correctly predicted to belong to the given class according to the ground truth, whereas a true negative (TN) represents a pixel that is correctly identified as not belonging to the given class. On the other hand, a false positive (FP) is an outcome where the model incorrectly predicts a pixel not belonging to a given class. A false negative (FN) is an outcome where the model incorrectly predicts the pixel belonging to a given class. Similarly, for tumor classification task, TP represents a tumor class that is correctly predicted to belong to the given class according to the ground truth whereas a TN represents a tumor class that is correctly identified as not belonging to the given class. By the same token, false positive (FP) is an outcome where the model incorrectly predicts a tumor class not belonging to a given class. A false negative (FN) is an outcome where the model incorrectly predicts the class belonging to a given class. Therefore, keeping different performance metrics used in brain tumor segmentation and classification literature are listed as follows.

Accuracy (ACC) measures the ability of a model in correctly identifying all class or pixels, no matter if it is positive or negative.

(1) $$ACC=\frac{TP+TN}{TP+TN+FP+FN}$$

Sensitivity (SEN) indicates the frequency of correctly predicted positive samples/pixels among all real positive/samples. It measures the models ability in identifying positive samples/pixels.

(2) $$SEN=\frac{TP}{TP+FN}$$

Specificity (SPE) is the proportion of actual negatives, which was predicted as the negative (or true negative). It tells the percentage of classes/pixels could not correctly identified.

(3) $$SPE=\frac{TN}{TN+FP}$$

Recall (RE) describes the completeness of the machine learning model’s positive predictions relative to the ground truth. It tells the percentage of classes/pixels annotated in our ground truth, are also included in model’s prediction.

(4) $$RE=\frac{TN}{TP+FN}$$

Precision (PR) also known as positive predictive value (PPV) describes how often the model predicting correct class/pixel. It tells the the correct proportion of models predicted positives.

(5) $$PR=\frac{TP}{TP+FP}$$

F1-Score is the most popular metric that combines both precision and recall. It represents harmonic mean of the two.

(6) $$F1score=2\frac{PR*RE}{\left(PR+RE\right)}$$

Intersection over union (IoU) also known as Jaccard index (JI) measures the percent overlap between the annotated ground truth mask and the model’s prediction output.

(7) $$IoU=\frac{TP}{TP+FP+FN}$$

Dice similarity coefficient (DSC) measures the spatial overlap between the ground truth tumor region and the model segmented region. A zero DSC value indicates no spatial overlap between the ground truth tumor region and model annotated result whereas a value indicates a indicating complete overlap between the two.

(8) $$DSC=\frac{TP}{\frac{1}{2}\left(2TP+FP+FN\right)}$$

Area under the curve (AUC) measure of the ability of a classifier to distinguish between classes and is used as a summary of the receiver characteristics curve and it is an area under true positive rate vs. false positive rate.

Similarity index (SI) refers to the similarity between the expert annotated ground truth and the model’s segmentation. It describes the similar identity between the input image and the detected tumor region.

(9) $$SI=\frac{2TP}{2TP+FP+FN}$$

## 5. Brain Tumor Segmentation Methods

Brain tumor imaging using techniques, such as MRI and CT, generate a significantly large number of images. Brain MRI scan of a single individual consists of several slices across the 3D anatomical view. Therefore, manual segmentation of brain tumors from magnetic resonance (MR) images is a challenging and time-consuming task. In addition, the artifacts introduced in the imaging process results in low-quality images that make the interpretation difficult. As a result, the manual brain MRI segment is susceptible for inter and intra observable variability. To alleviate these challenges and help radiologist, different automatic brain tumor segmentation techniques have been proposed in literature.

On these literature, authors have proposed an automated system for brain tumor segmentation techniques that provides objective, reproducible segmentation that are close to the manual results. These automated brain tumor segmentation can help to alleviate the difficulties associated with manually analyzing brain tumors. This will speed-up the brain image analysis process, improve diagnosis outcome, and make easy the follow-up of the disease through evaluating tumor progression [34].

In this section, among the proposed brain tumor segmentation techniques in the literature; region growing, machine learning, and deep learning based techniques will be surveyed to identify the experimental dataset, pre-processing, feature extraction, segmentation algorithm, and the reported performance.

### 5.1. Region-Based and Shallow Unsupervised Machine Learning Approach

One of the most commonly used segmentation techniques in automated image processing applications is region-based segmentation. Regions in an image are a group of connected pixels that satisfy certain homogeneity criteria, such as pixel intensity values, shape, and texture [35]. In a region-based segmentation the image is partitioned into dissimilar regions so that the desired region is located precisely [36]. The region-based segmentation takes into account the pixel values, such as gray level difference and variance, and spatial proximity of pixels, such as Euclidean distance and region compactness in grouping pixels together. In brain tumor segmentation, region growing, and clustering algorithms are the most commonly used region based segmentation technique.

Clustering-based segmentation is one of the powerful region based segmentation techniques where an image is partitioned into a number of disjoint groups. In clustering based segmentation pixels with high similarity categorized in a given region whereas dissimilar pixels categorized into different regions [37]. Clustering techniques, which are an unsupervised learning method, have been widely investigated in medical image segmentation. However, in this survey work some of the most popular clustering methods, such as k-means and its varieties [38,39,40,41,42,43,44], fuzzy c-means [38,39,41,45], subtractive clustering (SC), and hybrid techniques [46,47,48].

K-means clustering is an unsupervised machine learning algorithm and it is commonly used to segment a region of interest from the remaining part of an image. K-means has been extensively tested in brain tumor segmentation and has shown acceptable accuracy [48]. The minimal computational requirement [37,48], simplicity to implement on large dataset [49], adaptation to new examples, and guaranteed convergence are some of the advantages that makes K-means popular segmentation algorithm. However, k-means suffers with incomplete delineation of the tumor region [49], selection of the initial centroid is not optimum [37,43], and it is sensitive to outliers [48,50]. Due to these limitations a number of solutions have been proposed, including, evenly spreading the initial cluster centers (k-means++), hybridizing k-means with other clustering techniques [49], adaptively initializing cluster centers, such as adaptive k-means [43], modified adaptive k-means (MAKM), and histogram based k-means.

Fuzzy c-means works by assigning membership values to each of the pixels in an image corresponding to the centers of the clusters depending on a certain similarity criteria [51]. In fuzzy c-means (FCM) clustering objects can belong to more than one cluster based on its degree of membership. Therefore, in such a type of soft clustering technique, image pixels can occupy multiple clusters. As a result, compared to hard-clustering techniques such as k-means, FCM performs better on relatively noise free images. However, in medical images such as brain MRI that can be easily affected by unknown noises, the FCM performance is severely affected [52]. A number of researches have been performed to improve the limitation of FCM [53,54,55,56].

In region growing brain tumor segmentation, tissues including tumorous regions are partitioned based on certain similarity criterion, such as homogeneity, texture, sharpness, and gray levels. The technique starts by selecting an initial seed based on predefined methods. Then, the neighboring pixels are added progressively to the seed pixel [57]. The region growing based segmentation can properly segment regions with similar properties and spatially separated regions. However, it is sensitive to noise and influenced by the similarity criterion [57]. Therefore, it may end up with disconnected regions and results in a hole in the segmented region. Furthermore, finding a good initial seed is not an easy task [57]. Region growing and conventional unsupervised machine learning based brain tumor segmentation techniques proposed in literature are summarized in Table 3. The table indicates the brain MRI dataset used in the experiment, the centroid initialization techniques, the objective function, and the segmentation performance.

### 5.2. Supervised Shallow Machine Learning Based Approach

Supervised machine learning-based brain tumor segmentation approaches transformed the image segmentation problem into a tumorous pixel classification problem. The input vector for these supervised learning models consisted of different extracted features, and the output is a vector of desired classes for segmentation. In brain tumor segmentation, where tumor regions are often scattered all over the image, pixel classification rather than classical segmentation methods are often preferable [65]. Therefore, the traditional supervised machine learning algorithms have been used in the segmentation of a brain tumor from a head MRI scan [66,67,68,69,70,71,72,73,74,75,76].

In this section, as shown in Table 4, most relevant literature on brain tumor segmentation using traditional machine learning algorithms, such as support vector machine (SVM), artificial neural network (ANN), random forest (RF) are surveyed to identify data used, the pre-processing, feature extraction techniques, the classifier model, and whether or not post-processing is implemented.

### 5.3. Deep Learning-Based Approach

Deep learning methodologies produce automatic features that avoid or minimize the need for handcrafted features. In the deep learning-based brain tumor segmentation approach, the general strategy is to pass an image through the pipeline of deep learning building blocks and input image segmentation is performed depending on the deep features. In literature, there are a variety of deep learning techniques proposed for segmenting brain tumors. Some of such blocks contain deep convolutional neural networks (DCNNs), convolutional neural network (CNN), recurrent neural networks (RNNs), long short-term memory (LSTM), deep neural networks (DNNs), deep autoencoders (AEs), and generative adversarial networks (GANs). In this section, literature in terms of these building blocks, the dataset used, and the reported performance are presented as shown in Table 5.

## 6. Brain Tumor Classification Methods

Based on the WHO’s classification of central nervous system (CNS) tumors, there are more than 150 types of CNS tumors that are mainly categorized into primary and metastatic (secondary) tumors [99]. The primary tumors originate from the brain or the immediate surrounding tissues. Whereas, metastatic tumors arise from other body parts and migrate to the brain through the bloodstream. Metastatic tumors are considered cancerous or malignant, while primary tumors can be benign or malignant.

A biopsy is the existing gold standard procedure in brain tumor classification. However, it usually requires definitive brain surgery to take a sample [100,101]. On the other hand, an automated brain tumor classification from an MRI is non-invasive so that it avoids tumor sample taking procedure and it is safer. In addition, the machine learning-based brain tumor classification from an MRI scan can improve the diagnosis and treatment planning [101]. As a result, an automatic brain tumor classification from MRI images using machine or deep learning techniques is an active research area, and promising results have been achieved [100,102,103,104,105,106].

### 6.1. Conventional Machine Learning Based Approach

Machine learning is a paradigm where a machine is given a task where its performance improves with experience. Machine learning techniques are commonly grouped into three major types: supervised, unsupervised, and reinforcement learning [107]. Supervised learning is based on training a data sample from the data source with correct classification already assigned by domain experts, whereas, in unsupervised learning, the algorithm finds hidden patterns from the unlabeled data. On the other hand, reinforcement learning is carried out by making a sequence of decisions using reward signals. Therefore, the algorithm learns through receiving either rewards or penalties for the actions it performs [107]. Machine learning has been used in the classification of brain tumors from MRI images, and promising classification performance has been reported [108,109,110,111,112,113,114,115].

The traditional machine learning-based brain tumor classification techniques often consist of preprocessing, segmentation, feature extraction, and classification stages.

#### 6.1.1. Pre-processing

Brain MRI scans are significantly affected by different types of noises, including salt and pepper, Gaussian, Rician, and speckle noise [116,117,118]. These noises impose challenges in machine learning-based applications [117,119]. Therefore, obtaining high-quality image denoising is one of the important tasks in the pre-processing stage. Each method used in MRI denoising has its advantages and disadvantages. Several methods have been developed for reducing noises based on statistical property and frequency spectrum distribution [119]. In addition to denoising, tasks such as removing tags, smoothing the foreground region, intensity inhomogeneity correction, maintaining relevant edges, resizing, cropping, and skull stripping are part of pre-processing [110,111,112].

#### 6.1.2. Region of Interest (ROI) Detection

In an MRI brain scan, the segmentation task labels each voxel in an MRI image to specify its tissue type and anatomical structure [119]. The objective of ROI detection in tumor classification is to locate the tumor region from an MRI scan, improve the visualization, and allow quantitative measurements of image structures in the feature extraction stage [108,112]. Brain tumor segmentation can be performed in three different ways, namely, manual segmentation, semi-automatic segmentation, and fully automatic segmentation [119]. The autonomous brain segmentation techniques have been briefly discussed in Section 5.

#### 6.1.3. Feature Extraction

The feature extraction techniques are mathematical models based on various image properties. The different types of features include texture, brightness, contrast, shape, Gabor transforms, gray-level co-occurrence matrix (GLCM), and wavelet-based features [115,120], histogram of local binary patterns (LBP) [121]. On the other hand, recently, deep features that are obtained from deep neural networks such as CNN have been used as input to SVM classifier to classify brain tumors [122]. In brain tumor classification, it is customary to fuse several features from different extraction models to improve the discrimination power of the machine learning model [123]. Furthermore, feature selection is applied for dimensionality reduction.

#### 6.1.4. Classification

Different classification techniques have been proposed by many authors for identifying tumor types from brain images. Different authors have classified tumor into a variety of ways, for instance meningioma, glioma, and pituitary [109,121,122,124,125]; astrocytoma, glioblastoma, and oligodendrogliamo [112]; glioma tumor grades (I–IV) [113]; benign and malignant stages(I–IV) [126,127,128,129]; diffuse midline glioma, medulloblastoma, pilocytic astrocytoma, and ependymoma [102]; multifocal, multicentric, and gliomatosis [130]; ependymoma and pilocytic astrocytoma [120].

In brain tumor classification, the most commonly used classifiers are neural network [108,109,110,111,131], support vector machines (SVM) [108,115,124,127,128,129,130,132,133], K-nearest neighbor (KNN) [112,121,130,134], Adaboost [126], and hybrid models [113,135,136]. The neural network was implemented using different architectures, such as feedforward neural network [110,125], multilayer perceptron neural network [109,137], and probabilistic neural network (PNN) [111,131]. Support vector machine (SVM) was commonly implemented using three kernels, linear, homogeneous polynomial, and Gaussian radial basis function (RBF) [108,115]. In the KNNclassifier, the testing feature vector is classified by finding the k-nearest training neighbor, that is, the classifier does not use any model to match and is only based on memory. However, KNN uses different measurements such as euclidean distance, city block, cosine, and correlation to find the nearest distance between the testing and training class feature vectors [134].

A summary of recent shallow machine learning-based brain tumor classification techniques is given on Table 6.

### 6.2. Deep Learning Approach

Even though promising progress has been made in classifying brain tumors into their respective types from an MRI brain scan using shallow supervised machine learning algorithms, there are still challenges in classifying brain tumors from an MRI scan. These challenges are mainly due to the ROI detection, and extracting descriptive information using traditionally handcrafted feature extraction techniques is not efficient [122]. This inefficiency mainly arises due to the complex structure of brain anatomy and the high-density nature of the brain.

Unlike shallow machine learning algorithms, deep learning is based on learning data representations and hierarchical feature learning. In deep learning-based brain tumor classification, the deep learning models discover the descriptive information that optimally represents different brain tumors. This nature of deep learning transforms the brain tumor classification from handcrafted feature-driven into data-driven problem [103]. Among the deep learning models, a convolutional neural network (CNN) is widely used in brain tumor classification tasks, and a substantial result has been achieved[100].

In the reviewed literature, there are differences in the techniques used for the classification of brain tumors. The difference encompasses: (i) the dataset used for classification including tumor types, (ii) the implemented pre-processing and data augmentation techniques, (iii) whether or not the ROI segmentation was used as a prior step in the classification, (iv) whether a pre-trained or custom-designed deep learning model is used.

For instance, Badža and Barjaktarović[100] used publicly available contrast-enhanced T1-weighted brain tumor MRI scans [138]. The dataset contains meningioma, glioma, and pituitary brain tumor types scanned along with the three anatomical views, i.e., axial, sagittal, and coronal. The images were preprocessed using techniques, such as normalization and resizing. In addition, images in the dataset are augmented with 90^o^ rotation and vertical flipping to increase the training dataset. Furthermore, they used a custom-designed CNN model trained with Adam optimizer with a mini-batch size of 16 and tested with 10—fold cross-validation. The weights of the convolution layers are initialized using a Glorot initializer. The model performance was measure using sensitivity, specificity, accuracy, precision, recall, and F1-score. The sensitivity for meningioma, glioma, and pituitary is 89.8%, 96.2%, and 98.4%, respectively. The specificity of the model for meningioma, glioma, and pituitary is 90.2%, 95.5%, and 97.7%, respectively. Furthermore, the models’ overall accuracy, average precision, average recall, and F1-score are 95.4%, 94.81%, 95.07%, and 94.94%, respectively. The summary of this and other literature is presented on Table 7.

## 7. Discussion

This paper presented a thorough survey of techniques used in brain tumor segmentation and classification. The survey encompasses several traditional machine learning and deep learning-based methods with their quantitative performance. The conventional image segmentation techniques, that is, region growing and unsupervised machine learning used in brain tumor segmentation are presented in Table 3. The region growing with all other conventional image processing segmentation techniques is the earliest approach applied in brain tumor segmentation [161]. It is mainly affected by noises, poor image quality, and initial seed point. To overcome these challenges, an automatic seed point selection by optimization techniques and artificial intelligence-based seed point selection has been proposed [162]. In addition, it has a limitation in segmenting tumors that appear scattered across the brain. In the second generation segmentation techniques which are based on shallow unsupervised machine learning, such as fuzzy c-means and k-means grouping of pixels into more than one class has been achieved. However, these methods are also highly sensitive to noise. Therefore, through incorporating additional information and adaptively selecting the centroid, the segmentation performance of medical images can be improved [6]. In addition, the inherent ambiguous boundaries between normal tissues and brain tumors pose a significant challenge for conventional and clustering segmentation techniques. Therefore, to address this challenge, pixel-level classification-based segmentation techniques using traditional supervised machine learning have been proposed [70]. These methods are often accompanied by feature engineering, where the tumor descriptive pieces of information are extracted to train the model. Furthermore, the supervised machine learning segmentation output is further improved through post-processing [71,76].

**Figure 1:**
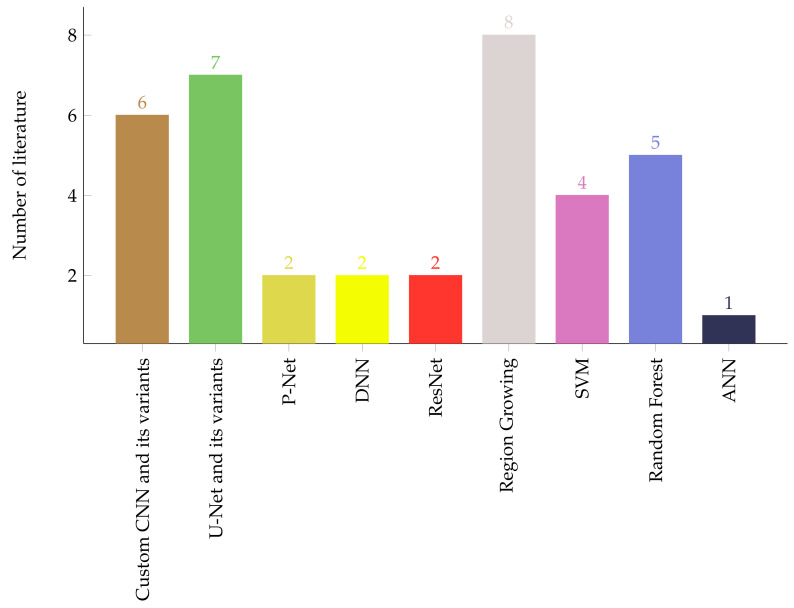
Number of brain tumor segmentation methods.

Nowadays, conventional image processing and shallow machine learning-based brain tumor segmentation techniques are becoming obsolete due to the advent of deep learning-based techniques. The deep learning-based approach performs an end-to-end tumor segmentation by passing an MRI image through the pipeline of its building blocks. These models often extract tumor descriptive information automatically and avoid the need for handcrafted features. However, the need for a large dataset to train the models and the difficulty in interpreting the models hinders their usage in medical fields [163]. In terms of segmentation performance, it is evident from Table 4 and Table 5 that the deep learning-based and supervised shallow machine learning-based with post-processing has comparable performances. Asummary of the number of brain tumor segmentation techniques surveyed in this is given on Figure 1.

**Figure 2:**
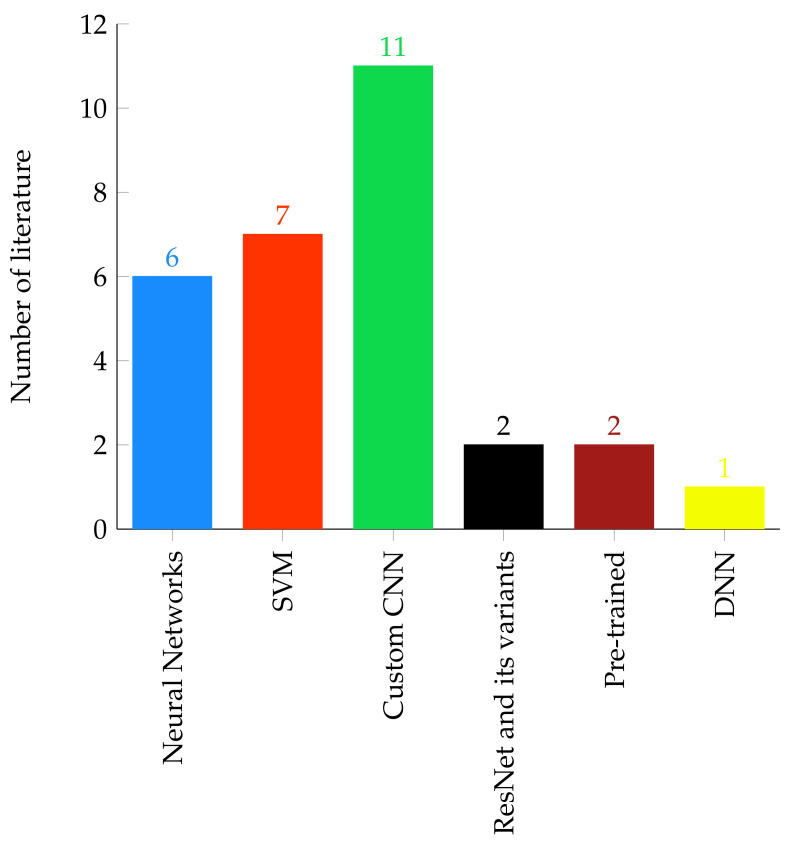
Number of brain tumor classification methods.

Aside from segmentation of brain tumor region from head MRI scan, classification of tumor into their respective histological type has great importance in diagnosis and treatment planning which actually requires biopsy procedure in today’s medical practice [158]. Several methods which encompass shallow machine learning and deep learning have been proposed for brain tumor classification. The conventional shallow machine learning algorithms often consist of preprocessing, ROI detection, and feature extraction. However, due to the inherent noise sensitivity of MRI image acquisition, variations in the shape, size, location, and contrast of tumor tissue cells, extracting descriptive information is a challenging task. Therefore, nowadays, deep learning techniques are becoming the state-of-the-art approach to classify different types of brain tumors, such as astrocytoma, glioma, meningioma, and pituitary. Several brain tumor classifications have been discussed in this survey, and a summary of the number of brain tumor classification techniques surveyed in this paper are given on Figure 2.

Several brain tumor datasets that are collected by researchers datasets and those that are available on repositories were used in the training and testing of brain tumor classification models. The publicly available dataset provided by J. Cheng et al. [138], which contains meningioma, glioma, and pituitary tumor in T1-WC MRI-images is one of the most commonly used datasets in the training and testing classifier models. Using this dataset, Gumaei, A. et al. [125] has achieved a classification accuracy of 94.23% using a regularized extreme learning machine, while the Kokkalla, S. et al. [153] have reported a classification accuracy of 99.69% using custom modified deep-dense inception residual network (DDIRNet). These results indicate that the deep learning-based model outweighs the shallow machine learning-based techniques for this particular dataset.

### Challenges in Automatic Brain Tumor Segmentation and Classification

The development of autonomous brain tumor segmentation and classification models using MRI images is still a challenging task. The challenges are due to several constraints including the effect of different types of noises embedded in the brain MRI images [116,117,118], motion and metal artifacts during image acquisition [164], low-resolution MRI images [165], and lack of deep learning models interpretability and transparency [166,167].

One of the most common challenges in machine learning-based brain tumor segmentation and classification is the noisiness of an MRI image. Therefore, noise estimation and denoising MRI images is a crucial pre-processing task for improving the accuracy of brain tumor segmentation and classification models. Therefore, several techniques have been proposed for denoising MRI images, such as modified iterative grouping median filter [118], Wiener filter and wavelet transform [168], non-local means [169], and deep learning-based approaches [170,171]. However, a robust denoising technique for MRI images is still challenging and the pursuit to obtain an efficient denoising technique has been an active research area [170]. Similarly, motion, metal, and other artifacts are also a source of challenge to the robustness of machine learning-based brain tumor segmentation and classification. Recently, deep learning-based solutions for minimizing the effects of these artifacts have been proposed [164,172]. MRI provides a high fidelity brain scan image compared to other imaging techniques. However, post-acquisition image processing techniques, including deep learning-based methods have been used to increase the resolution of MR images so that the efficiency of autonomous brain tumor segmentation and classification models improved[165,173]. The other major challenge is the lack of deep models’ interpretability, and often they are perceived as black-box. As a result, attaining any evidence regarding the process they perform is difficult. However, the transparency and interpretability of deep learning techniques are crucial for the complete integration into medical diagnosis [166].

## 8. Conclusions

Automating the brain tumor segmentation and classification task has tremendous benefits in improving the diagnosis, treatment planning, and follow-up of patients. Through applying various techniques, including conventional image processing, shallow machine learning, and deep learning techniques, undeniable progress have been achieved in automating brain tumor segmentation and classification tasks. However, building a fully autonomous system that can be used on clinical floors is still a challenging task.

Compared to region-growing and shallow machine learning algorithms, automating the brain tumor segmentation and classification using deep learning techniques have huge benefits. This is mainly due to the powerful feature learning ability of deep learning techniques. In addition, as can be shown in Figure 1 and Figure 2, deep learning-based brain tumor segmentation and classification techniques are becoming the most active research area. In this paper, a comprehensive survey on region growing, shallow machine learning, and deep learning-based brain tumor segmentation and classification methods are presented. These methods are structurally categorized and summarized to give an insight to the reader of the dataset used, pre-processing, feature extraction, segmentation, classification, post-processing, and the reported model performances in the literature. Furthermore, the pros and cons of the methods and the model evaluation metrics have been discussed.

## Figures and Tables

**Table 1:** Survey literature on brain tumor segmentation and classification techniques.

Author and Publication Year	Strength	Limitation
Sharma and Shukla [29] 2021	Thresholding, conventional supervised and unsupervised based segmentation techniques are briefly described.	A very shallow discussion on deep learning based brain tumor segementation and classification. The performances of the surveyed literature are not inculded.
Rao and Karunakara [30] 2021	Differnt brain tumor segmentation techniques that includes thresholding, region growing, atlas, deep learning, and conventional supervised and unsupervised machine learning based have been discussed. The performances of tumor classification techniques were clearly presented.	Chronologically majority of the reviewed papers on brain tumor classification are from 2019 and earlier. Except two literature that are published on 2020. The segmentation and classification techniques are not clearly distingushed while presenting their performce metrices.
Magadza and Viriri [31] 2021	Deep learning based brain tumor segmentation techniques are presented in detail; including, their building blocks	The survey does not include brain tumor classification techniques and conventional machine learning based tumor classification and segmentation techniques. Segmentation performce of top performing models on BRATs dataset is provided.
Tiwari et al. [32] 2020	A detailed hierarchical classification of brain tumor presented. A brain tumor segmentation techniques, including: those based on thresholding, conventional supervised and unsupervised machine learning, and deep learning are discussed. Conventional machine learning and deep learning based brain tumor classification techniques are surveyed.	Chronologically, literature earlier than and including 2019 are reviewed. A small number of deep learning based brain tumor segmentation and classification literature are reviewed.
Kumari and Saxena [33] 2018	A limited literature that encompases different segmentation techniques including thresholding, deep learning, and supervised and unsupervised machine learning techniques were reviewed.	Rather than reviewing literature on brain tumor classification, the paper only discusses the pros and cons of the classification algorithms. Aside from the limited discussion on brain tumor segmentation techniques, the review did not include the performance of proposed techniques. Furthermore, the review work incorporates literature before 2018.

**Table 2:** Inclusion and exclusion criteria for paper selection.

IC	EC
IC1: Paper must be peer reviewed.	EC1: Duplicate studies in different databases.
IC2: Journals on which papers published must be either scopus or web of science indexed	EC2: Study that uses imaging techniques other than MRI.
IC3: The paper should use only MRI brain images	EC3: Study which is less cited by other peer reviewed papers.
	EC4: MSc and PhD papers.
	EC5: Case study papers.

**Table 3:** Region growing and shallow unsupervised machine learning based brain tumor segmentation.

Paper	Dataset	Segmentation Technique	Objective Function	Performance
[58]	BRATS 2015 BRATS-MICCAI	Multi-level thresholding with level-set segmentation	Euclidean distance	JI 81.94%, DSC 89.91%
[48]	https://radiopaedia.org/ (accessed on 3 May 2021)	K-means and FCM	Euclidean distance	ACC 56.4 %
[43]	BRATS	K-means with histogram peaks centroid initialization	Euclidean distance	-
[39]	BRATS	Patch based k-means with FCM	Euclidean distance	SI 91%
[42]	BRATS 2012	Random	Sum of Squared Error	DSC 91%
[44]	MRI images collected by authors	Bi-secting (No initialization)	Sum of Squared Error	ACC 83.05%
[59]	BRATS	Force Clustering	Distance (in pixels)	-
[60]	BRATS 2017	Random	Euclidean distance	DSC 62.5%
[61]	MRI images collected by authors	DPSO ^1^	Euclidean distance	ACC 99.98%, SEN 95.02%, SPE 99.92% DSC 93.09%
[62]	MRI images collected by authors	FCM preceded by gross tumor volume segmentation with random centroid intialization	Inter-cluster variance	DSC 95.93 ± 4.23%, JI 92.81 ± 6.56%, SPE 95.31 ± 6.56%, SEN 98.09 ± 1.75%
[63]	MRI images collected by authors	DWT ^2^ based genetic algorithm (GA)	fitness function variance	ACC 97%
[64]	MRI images collected by authors	semi-automatic cellular automata seeded segmentation with morphological post-processing	pixel similarity function	DSC 90.88 ± 4.19%, JI 84.11 ± 6.74%, SPE 99.99 ± 0.01%, SEN 91.20 ± 7.00%

^1^ Darwinian Particle Swarm Optimization, ^2^ Discrete Wavelet Transform.

**Table 4:** Summary of a shallow machine learning based segmentation.

Paper	Dataset	Preprocessing	Features	Model	Post- Processing	Performance
[66]	Clinically collected MRI	N4ITK	deep features from CNN	SVM	-	DSC 88%, SEN 89%, PR 83%
[67]	Clinically collected MRI	Registration	Intensity texture	Multi- kernel SVM	Region growing	TP 98.9%, FP 4.5%, FN 3.1%
[68]	BRATS 2013	N4ITK, histogram matching, SLIC ^1^	Gray statistical, GLCM	SVM	-	DSC 86.12%, SEN 79.69%, SPE 99.48%
[70]	BRATS 2015	-	Intensity, texture	ANN, SVM	-	SVM: DSC 88.7%, IOU 79.7%, ANN: DSC 90.79%, IOU 83.1%
[71]	BRATS 2015, [77,78,79]	-	Dual pathway tree based features	ccRF ^2^	mpAC ^3^	DSC 89%, SPE 90%, SEN 85%
[72]	BRATS 2012	registration, normalization	intensity, similarity, blobness	RF	Independent connected component analysis	DSC 96.5%
[74]	[80]	N4ITK, normalization, histogram matching	intensity, gradient, context	RDF ^4^	morphological filtering	DSC 86.41%, SEN 82%, PR 92.92%
[75]	BRATS 2015	noise removal, enhancement	first higher order features, texture	RF	morphological other filtering	DSC 98.4%, SEN 97.9%, SPE 80.7%, ACC 97.7%
[76]	BRATS 2015	histogram enhancement	Gabor wavelet, intensity	RF	morphological other filtering	DSC 85.5%, SEN 77.1%, SPE 99.3%

^1^ Simple Linear Iterative Clustering, ^2^ Concatenated and Connected Random Forest, ^3^ Multiscale Patch Driven Active Contour, ^4^ Random Decision Forest.

**Table 5:** Summary of deep learning based brain tumor segmentation techniques.

Paper	Dataset	Preprocessing	Model Architecture	Performance
[81]	BRATS 2013 & 2015	bias field correction, intensity and patch normalization, augmentation	Custom CNN	DSC 88%, SEN 89%, PR 87%
[82]	BRATS 2013	intensity normalization, augmentation	HCNN + CRF-RRNN ^1^	SEN 95%, SPE 95.5%, PR 96.5%, RE 97.8%, ACC 98.6%
[83]	BRATS 2015	Z-score normalization on the image,	Residual Network+ Dilated convolution RDM-Net ^2^	DSC 86%
[84]	BRATS 2015	Z-score normalization	Stack Multi-connection Simple Reducing_Net (SMCSRNet)	DSC 83.42%, PR 78.96%, SEN 90.24%
[85]	BRATS 2019	-	Ensemble of a 3D-CNN and U-net	DSC 90.6%
[86]	BRATS 2015	Bias correction, intensity normalization	Two-PathGroup-CNN (2PG-CNN)	DSC 89.2%, PR 88.22%, SEN 88.32%
[87]	BRATS 2018	-	Hybrid two track U-Net (HTTU-Net)	DSC 86.5%, SEN 88.3%, SPE 99.9%
[88]	BRATS 2015	-	P-Net with bounding box and image specific fine tunning (BIFSeg)	DSC 86.29%
[89]	ADNI	denoising, Skull stripping, sub-sampling	Multi-scale CNN (MSCNN)	ACC 90.1%
[90]	BRATS 2017	Intensity normalization, resizing, Bias field correction	Cascaded 3D U-nets	DSC 89.4%
[91]	BRATS 2015 & 2017	Down sampling	3D Center-crop Dense Block	BRATS 2015: DSC 88.4%, SEN 83.8% BRATS 2017: DSC 88.7%, SEN 84.3%
[92]	BRATS 2018 & 2019	Z-score normalization, cropping	3D FCN ^3^	BRATS 2018: DSC 90%, SEN 90.3, SPE 99.48%; BRATS 2019: DSC 89%, SEN 88.3%, SPE 99.51%
[93]	BRATS 2018	intensity normalization, removing 1% of highest & lowest intensity	DCNN (Dense-MultiOCM ^4^)	BRATS 2018: DSC 86.2%, SEN 84.8 %, SPE 99.5%
[94]	TCIA	Image cropping, padding, resizing, intensity normalization	U-Net	DSC 84%, SEN 92%, SPE 92%, ACC 92%
[95]	BRATS 2013, 2015, 2018	-	AFPNet ^5^ + 3D CRF	BRATS 2013 DSC 86%, BRATS 2015 DSC 82%, BRATS 2018 86.58%
[96]	BRATS 2015, 2017	z-score normalization	Inception-based U-Net + up skip connection + cascaded training strategy	DSC 89%, PR 78.5%, SEN 89.5%
[97]	BRATS 2015, BrainWeb	cropping, z-score normalization, min-max normalization (BrainWeb)	Tripple intersecting UNets (TIU-Net)	BRATS 2015: DSC 85%, BrainWeb DSC 99.5%
[98]	BRATS 2015	-	LSTM multi-modal UNet	DSC 73.09%, SEN 63.76%, PR 89.79%

^1^ Heterogeneous CNN + Conditional Random Fields-Recurrent Regression based Neural Network, ^2^ Deep Residual Dilate Network with Middle Supervision, ^3^ Fully Convolutional Neural Network, ^4^ OCcipito Module, ^5^ Atrous-Convolution Feature Pyramid.

**Table 6:** Summary of conventional ML based brain tumor classification techniques.

Paper	Dataset	Preprocessing	ROI Detection	Feature Extraction	Classifier	Tumor Types	Performance
[108]	Local dataset	Median and weiner filter	k-means modified FCM	shape features, statistical features	ANN	Benign malignant stage (I-IV)	SPE 100%, SEN 98%, ACC 97.73%, BER 0.0294
[109]	[138]	Median and weiner filter	manually	2-D DWT 2-D Gabor feature	ANN	Glioma (GL), Meningioma (MG) Pituitary tumor (PT)	overall ACC 91.9%, SPE (GL) 96.29%, SPE (MG) 96%, SPE (PT) 96.2%, SEN (GL) 95.1%, SEN(MG) 86.97%, SEN(PT) 91.24%
[110]	Local dataset	resizing skull removing	Canny	Gabor filter, GLCM DWT	ANN	Benign and malignant stage (I-IV)	SPE 98.5%, SEN 99.1%, ACC 98.9%
[139]	Local dataset	resizing	-	PCA ^1^	PNN	Benign malignant stage	SPE 100%, SEN 92.3%, ACC 97.4%
[112]	TCIA	resizing, cropping, median filtering	morphological, watersheed	shape features	KNN	Astrocytoma Glioblastoma Oligodendroglioma	ACC 89.5%
[115]	Local dataset	wavelets	thresholding	DWT coeficients statistical features	SVM	Benign malignant	ACC (linear) 92%, ACC (kernel) 99%
[134]	BRATS and Local dataset	enhancement median filter	Morphological	GLCM features	SVM	Benign malignant	BRATS: SVM (linear):SPE 100%, SEN 72%, ACC 82.5% SVM (Quadratic):SPE 73.3%, SEN 88%, ACC 82.5% SVM (RBF): SPE 100%, SEN 76%, ACC 85% Clinical: SVM (linear):SPE 60%, SEN 76%, ACC 68% SVM (Quadratic):SPE 88%, SEN 100%, ACC 94% SVM (RBF): SPE 100%, SEN 92%, ACC 96%
[120]	Local dataset			Gabor transform texture wavelet	SVM	Ependymoma Pilocytic Astrocytoma	SPE 80%, SEN 93%, ACC 88%, AUC 0.86
[140]	BRATS-2015	wavelet filters, inhomogeneity correction	edge detection, morphological operations	shape, texture, intensity	PSO ^2^-SVM	Benign, malignant	SPE 94.8%, SEN 100%
[136]	-	median filtering skull removing	thresholding	GLCM	GA-SVM	Benign, malignant	-
[130]	REMBRANDT	-	-	texture features	SVM	Multifocal, Multicentric, Gliomatosis	PR 90%, SEN 90%, ACC 90%, F1-Score 90%
[133]	Local dataset	Image fusion with contourlet transform	Otsu’s thresholding	curvlet transform GLCM features	SVM	Benign, Malignant	ACC 93%
[125]	[138]	min-max normalization,	-	NGIST features	RELM ^3^	Meningioma, Glioma, Pituitary	ACC 94.23%
[126]	Local dataset	median filter	thresholding	GLCM texture features	Adaboost	Benign, Malignant	SPE 62.5%, SEN 88.25%, ACC 89.90%
[127]	Local dataset	resizing enhancement	morphological, thresholding	GLCM statistical texture features	SVM	Benign, Malignant	SPE 62.5%, SEN 88.25%, ACC 89.90%
[128]	Local dataset	noise removal, enhancement	Expectation maximization, levelset	GA, statistical features	SVM	Benign, Malignant	SPE 100%, SEN 98%, ACC 98.30%
[124]	[138]	down sampling Gabor filter	-	statistical features	SVM	Meningioma, Glioma, Pituitary	Meningioma: SVM (linear):RE 0.63, PR 0.66, ACC 82.38% SVM (poly):RE 0.62,Pr. 0.73, ACC 84.33% Glioma: SVM (linear):RE 0.82, PR 0.82, ACC 83.01% SVM (poly):RE 0.88, PR 0.79, ACC 84.01% Pituitary: SVM (linear):RE 0.94, PR 0.90, ACC 95.27% SVM (poly):RE 0.91,PR 0.94, ACC 95.43%
[122]	Kaggle Brain Tumor Detection 2020	cropping, resizing using bicubic interpolation	-	Deep features from pretrained CNN	SVM	Meningioma, Glioma, Pituitary	ACC 90.19%

^1^ Principal Component Analysis, ^2^ Particle Swarm Optimization, ^3^ Regularized Extreme Learning Machine.

**Table 7:** Summary of deep learning based brain tumor classification techniques.

Paper	Dataset	Preprocessing	Classifier Model	Tumor Types	Performance
[100]	[138]	normalization, resizing, augmentation	Custom CNN model	Meningioma, Glioma, Pituitary	ACC 91.9%, precision 94.81%, RE 95.07%, F1-score 94.94%, SPE(GL) 96.2%, SPE(MG) 92%, SPE(PT) 97.7%, SEN(GL) 96.2%, SEN(MG) 89.8%, SEN(PT) 98.4%
[141]	[78,142]	Augmentation using GAN	Multi-stream 2D-CNN model	Glioma subtypes: Isocitrate dehydrogenase 1 mutation (IDH1), & IDH1 wild-type	mean ACC 88.82% mean SEN 81.81% mean SPE 92.17%
[143]	[138,144]	resizing augmentation	Custom CNN model	Meningioma, Glioma & Pituitary and Glioma (grade:II-IV)	MG: PR 95.8%, SEN 95.5%, SPE 98.7%, ACC 97.54%, GL: PR 97.2%, SEN 94.4%, SPE 95.1%, ACC 95.81%, PT: PR 95.2%, SEN 93.4%, SPE 97%, ACC 96.89% Grade II: PR 100%, SEN 100%, SPE 100%, ACC 100%, III: PR 100%, SEN 95%, SPE 100%, ACC 95%, IV:PR 96.3%, PR 100%, SEN 95%, SPE 100%, ACC 95%SEN 100%, SPE 98%, ACC 100%
[145]	[138]	-	CNNBCN ^1^	Meningioma, Glioma& Pituitary	ACC 95.49%
[146]	[138]	-	BayesCap: captures prediction uncertainity	Meningioma, Glioma& Pituitary	mean ACC 73.9% CI ^2^:(73.4%, 74.4%)
[147]	[138]	Image rotation, resizing	AutoML ^3^	Meningioma, Glioma & Pituitary	MG: PR 94.51%, SEN 87.76%, SPE 98.7%, ACC 96.29%, F1-Score 91.01%, MCC ^4^ 88.77%, G-Mean 96.09% GL: PR 96.97%, SEN 95.32%, SPE 96.88%, ACC 96.08%, F1-Score 96.14%, MCC 92.17%, G-Mean 96.09% PT: PR 91.61%, SEN 99.24%, SPE 96.27%, ACC 97.14%, F1-Score 95.27%, MCC 93.38%, G-Mean 97.75%
[148]	[138]	-	Iception-V3 DensNet201	Meningioma, Glioma& Pituitary	Iception-V3: ACC 99.34% DensNet201: ACC 99.51%
[149]	[138]	augmentation, contrast- stretching	AlexNet, GoogleNet & VGG16 ^5^	Meningioma, Glioma& Pituitary	AlexNet: ACC 95.46% GoogleNet: ACC 98.04% VGG16 98.69%
[150]	[138]	-	ConvCaps	Meningioma, Glioma& Pituitary	ACC 93.5%
[151]	[138]	flipping, patching	CapsulNet	Meningioma, Glioma& Pituitary	MG: PR 85%, RE 94%, F1-Score 94, %GL: PR 85%, RE 94%, F1-Score 94%, PT: PR 85%, RE 94%, F1-Score 94%
[152]	[138]	-	G-ResNet	Meningioma, Glioma& Pituitary	ACC 95%
[153]	[138]	-	DDIRNet ^6^	Meningioma, Glioma& Pituitary	ACC 99.69%, PR 99.6%, RE 99.4%, F1-score 99.4%
[103]	[138]	-	Multiscale CNN	Meningioma, Glioma& Pituitary	ACC 97.3%
[154]	[155]	DWT	DNN	Meningioma, Glioma& Pituitary	ACC 96.15%, PR 94.12%, AUC 98.75%,F1-score 96.97%, RE 100%
[156]	[138]	-	Custom CNN model	Meningioma, Glioma& Pituitary	ACC 84.19%
[157]	BraTS 2018 & 2019	-	Pre-trained DenseNet201	HGG ^7^ & LGG ^8^	HGG: ACC 99.8%, LGG: ACC 99.3%
[158]	[138], [144,159]	-	Custom CNN model	Class 1: Normal, Metastatic, Meningioma, Glioma& Pitiutary Class 2: Grade II, III & IV	Class 1: ACC 92.66% Class 2: ACC 98.14%
[160]	BraTS 2019	-	Custom CNN model	Astrocytoma, Glioblastoma, Oligodendrogloma,	Class 1: ACC 92.66% Class 2: ACC 98.14%
[94]	TCIA	cropping, padding, resizing, normalization	VGG16	Grade II & III	ACC 89%, SEN 87%, SPE 92%

^1^ Convolutional Neural Network based on Complex Networks, ^2^ Confidence Interval, ^3^ Automated Machine Learning, ^4^ Matthew’s Correlation Coefficient, ^5^ Visual Geometry Group, ^6^ Deep Dense Inception Residual Network, ^7^ High Grade Glioma, ^8^ Low Grade Glioma.

## Data Availability

Not applicable.

## References

[1] Afework Y.K., Debelee T.G. (2020). Detection of Bacterial Wilt on Enset Crop Using Deep Learning Approach. Int. J. Eng. Res. Afr..

[2] Debelee T.G., Schwenker F., Ibenthal A., Yohannes D. (2019). Survey of deep learning in breast cancer image analysis. Evol. Syst..

[3] Debelee T.G., Kebede S.R., Schwenker F., Shewarega Z.M. (2020). Deep Learning in Selected Cancers’ Image Analysis—A Survey. J. Imaging.

[4] Debelee T.G., Amirian M., Ibenthal A., Palm G., Schwenker F. (2018). Classification of Mammograms Using Convolutional Neural Network Based Feature Extraction. Lecture Notes of the Institute for Computer Sciences, Social Informatics and Telecommunications Engineering.

[5] Debelee T.G., Gebreselasie A., Schwenker F., Amirian M., Yohannes D. (2019). Classification of Mammograms Using Texture and CNN Based Extracted Features. J. Biomimetics Biomater. Biomed. Eng..

[6] Debelee T.G., Schwenker F., Rahimeto S., Yohannes D. (2019). Evaluation of modified adaptive k-means segmentation algorithm. Comput. Vis. Media.

[7] Kebede S.R., Debelee T.G., Schwenker F., Yohannes D. (2020). Classifier Based Breast Cancer Segmentation. J. Biomimetics Biomater. Biomed. Eng..

[8] Megersa Y., Alemu G. Brain tumor detection and segmentation using hybrid intelligent algorithms. Proceedings of the AFRICON 2015.

[9] Roberts T.A., Hyare H., Agliardi G., Hipwell B., d’Esposito A., Ianus A., Breen-Norris J.O., Ramasawmy R., Taylor V., Atkinson D. (2020). Noninvasive diffusion magnetic resonance imaging of brain tumour cell size for the early detection of therapeutic response. Sci. Rep..

[10] Villanueva-Meyer J.E., Mabray M.C., Cha S. (2017). Current Clinical Brain Tumor Imaging. Neurosurgery.

[11] Rosenbloom M.J., Pfefferbaum A. (2008). Magnetic resonance imaging of the living brain: Evidence for brain degeneration among alcoholics and recovery with abstinence. Alcohol Res. Health J. Natl. Inst. Alcohol Abus. Alcohol..

[12] Noback C.R., Strominger N.L., Demarest R.J., Ruggiero A.D. (2005). The Human Nervous System: Structure and Function.

[13] Louis D.N., Ohgaki H., Wiestler O.D., Cavenee W.K. (2007). WHO Classification of Tumors of the Central Nervous System.

[14] Kayode A.A., Shahzadi A., Akram M., Anwar H., Kayode O.T., Akinnawo O.O., Okoh S.O. (2020). Brain Tumor: An overview of the basic clinical manifestations and treatment. Glob. J. Cancer Ther..

[15] Johnson D.R., Guerin J.B., Giannini C., Morris J.M., Eckel L.J., Kaufmann T.J. (2017). 2016 Updates to the WHO Brain Tumor Classification System: What the Radiologist Needs to Know. RadioGraphics.

[16] Roth P., Pace A., Rhun E.L., Weller M., Ay C., Moyal E.C.J., Coomans M., Giusti R., Jordan K., Nishikawa R. (2021). Neurological and vascular complications of primary and secondary brain tumours: EANO-ESMO Clinical Practice Guidelines for prophylaxis, diagnosis, treatment and follow-up. Ann. Oncol..

[17] Buckner J.C., Brown P.D., O’Neill B.P., Meyer F.B., Wetmore C.J., Uhm J.H. (2007). Central Nervous System Tumors. Mayo Clinic Proceedings.

[18] Smithuis R. Neuroradiology: Brain Index. https://radiologyassistant.nl/neuroradiology/brain.

[19] Alves A.F.F., de Arruda Miranda J.R., Reis F., de Souza S.A.S., Alves L.L.R., de Moura Feitoza L., de Souza de Castro J.T., de Pina D.R. (2020). Inflammatory lesions and brain tumors: Is it possible to differentiate them based on texture features in magnetic resonance imaging?. J. Venom. Anim. Toxins Incl. Trop. Dis..

[20] Kasban H. (2015). A Comparative Study of Medical Imaging Techniques. Int. J. Inf. Sci. Intell. Syst..

[21] Ammari S., Pitre-Champagnat S., Dercle L., Chouzenoux E., Moalla S., Reuze S., Talbot H., Mokoyoko T., Hadchiti J., Diffetocq S. (2021). Influence of Magnetic Field Strength on Magnetic Resonance Imaging Radiomics Features in Brain Imaging, an In Vitro and In Vivo Study. Front. Oncol..

[22] Rajasekaran K.A., Gounder C.C. (2018). Advanced Brain Tumour Segmentation from MRI Images. High-Resolution Neuroimaging—Basic Physical Principles and Clinical Applications.

[23] Foltz W.D., Jaffray D.A. (2012). Principles of Magnetic Resonance Imaging. Radiat. Res..

[24] Hornark J.P. The Basics of MRI. http://www.cis.rit.edu/htbooks/mri.

[25] Mustafa W.F., Abbas M., Elsorougy L. (2020). Role of diffusion-weighted imaging in differentiation between posterior fossa brain tumors. Egypt. J. Neurol. Psychiatry Neurosurg..

[26] Salama G.R., Heier L.A., Patel P., Ramakrishna R., Magge R., Tsiouris A.J. (2018). Diffusion Weighted/Tensor Imaging, Functional MRI and Perfusion Weighted Imaging in Glioblastoma—Foundations and Future. Front. Neurol..

[27] Fink J.R., Muzi M., Peck M., Krohn K.A. (2015). Multimodality Brain Tumor Imaging: MR Imaging, PET, and PET/MR Imaging. J. Nucl. Med..

[28] Luo Q., Li Y., Luo L., Diao W. (2018). Comparisons of the accuracy of radiation diagnostic modalities in brain tumor. Medicine.

[29] Sharma P., Shukla A.P. A Review on Brain Tumor Segmentation and Classification for MRI Images. Proceedings of the 2021 International Conference on Advance Computing and Innovative Technologies in Engineering (ICACITE).

[30] Rao C.S., Karunakara K. (2021). A comprehensive review on brain tumor segmentation and classification of MRI images. Multimed. Tools Appl..

[31] Magadza T., Viriri S. (2021). Deep Learning for Brain Tumor Segmentation: A Survey of State-of-the-Art. J. Imaging.

[32] Tiwari A., Srivastava S., Pant M. (2020). Brain tumor segmentation and classification from magnetic resonance images: Review of selected methods from 2014 to 2019. Pattern Recognit. Lett..

[33] Kumari N., Saxena S. Review of Brain Tumor Segmentation and Classification. Proceedings of the 2018 International Conference on Current Trends towards Converging Technologies (ICCTCT).

[34] Meier R., Knecht U., Loosli T., Bauer S., Slotboom J., Wiest R., Reyes M. (2016). Clinical Evaluation of a Fully-automatic Segmentation Method for Longitudinal Brain Tumor Volumetry. Sci. Rep..

[35] Pohle R., Toennies K.D., Sonka M., Hanson K.M. (2001). Segmentation of medical images using adaptive region growing. Medical Imaging 2001: Image Processing.

[36] Dey N., Ashour A.S. (2018). Computing in Medical Image Analysis. Soft Computing Based Medical Image Analysis.

[37] Dhanachandra N., Manglem K., Chanu Y.J. (2015). Image Segmentation Using K -means Clustering Algorithm and Subtractive Clustering Algorithm. Procedia Comput. Sci..

[38] Hooda H., Verma O.P., Singhal T. Brain tumor segmentation: A performance analysis using K-Means, Fuzzy C-Means and Region growing algorithm. Proceedings of the 2014 IEEE International Conference on Advanced Communications, Control and Computing Technologies.

[39] Bal A., Banerjee M., Sharma P., Maitra M. Brain Tumor Segmentation on MR Image Using K-Means and Fuzzy-Possibilistic Clustering. Proceedings of the 2018 2nd International Conference on Electronics, Materials Engineering & Nano-Technology (IEMENTech).

[40] Kumar D.V., Krishniah V.J.R. (2018). Segmentation of Brain Tumor Using K-Means Clustering Algorithm. J. Eng. Appl. Sci..

[41] Selvakumar J., Lakshmi A., Arivoli T. Brain tumor segmentation and its area calculation in brain MR images using K-mean clustering and Fuzzy C-mean algorithm. Proceedings of the IEEE-International Conference on Advances in Engineering, Science And Management (ICAESM-2012).

[42] Shanker R., Singh R., Bhattacharya M. Segmentation of tumor and edema based on K-mean clustering and hierarchical centroid shape descriptor. Proceedings of the 2017 IEEE International Conference on Bioinformatics and Biomedicine (BIBM).

[43] Kaur N., Sharma M. Brain tumor detection using self-adaptive K-means clustering. Proceedings of the 2017 International Conference on Energy, Communication, Data Analytics and Soft Computing (ICECDS).

[44] Mahmud M.R., Mamun M.A., Hossain M.A., Uddin M.P. Comparative Analysis of K-Means and Bisecting K-Means Algorithms for Brain Tumor Detection. Proceedings of the 2018 International Conference on Computer, Communication, Chemical, Material and Electronic Engineering (IC4ME2).

[45] Shasidhar M., Raja V.S., Kumar B.V. MRI Brain Image Segmentation Using Modified Fuzzy C-Means Clustering Algorithm. Proceedings of the 2011 International Conference on Communication Systems and Network Technologies.

[46] Agrawal R., Sharma M., Singh B.K. (2019). Segmentation of Brain Tumour Based on Clustering Technique: Performance Analysis. J. Intell. Syst..

[47] Pitchai R., Supraja P., Victoria A.H., Madhavi M. (2020). Brain Tumor Segmentation Using Deep Learning and Fuzzy K-Means Clustering for Magnetic Resonance Images. Neural Process. Lett..

[48] Almahfud M.A., Setyawan R., Sari C.A., Setiadi D.R.I.M., Rachmawanto E.H. An Effective MRI Brain Image Segmentation using Joint Clustering (K-Means and Fuzzy C-Means). Proceedings of the 2018 International Seminar on Research of Information Technology and Intelligent Systems (ISRITI).

[49] Abdel-Maksoud E., Elmogy M., Al-Awadi R. (2015). Brain tumor segmentation based on a hybrid clustering technique. Egypt. Informatics J..

[50] Mannor S., Jin X., Han J., Jin X., Han J., Jin X., Han J., Zhang X. (2011). K-Medoids Clustering. Encyclopedia of Machine Learning.

[51] Bezdek J.C., Hall L.O., Clarke L.P. (1993). Review of MR image segmentation techniques using pattern recognition. Med. Phys..

[52] Blessy S.A.P.S., Sulochana C.H. (2014). Performance analysis of unsupervised optimal fuzzy clustering algorithm for MRI brain tumor segmentation. Technol. Health Care.

[53] Arakeri M.P., Reddy G.R.M. (2011). Efficient Fuzzy Clustering Based Approach to Brain Tumor Segmentation on MR Images. Communications in Computer and Information Science.

[54] Dubey Y.K., Mushrif M.M. (2016). FCM Clustering Algorithms for Segmentation of Brain MR Images. Adv. Fuzzy Syst..

[55] Badmera M.S., Nilawar A.P., Karwankar A.R. Modified FCM approach for MR brain image segmentation. Proceedings of the 2013 International Conference on Circuits, Power and Computing Technologies (ICCPCT).

[56] Sheela C.J.J., Suganthi G. (2019). Automatic Brain Tumor Segmentation from MRI using Greedy Snake Model and Fuzzy C-Means Optimization. J. King Saud Univ. Comput. Inf. Sci..

[57] Wang Y. (2010). Tutorial: Image Segmentation.

[58] Rajinikanth V., Fernandes S.L., Bhushan B., Harisha, Sunder N.R. Segmentation and Analysis of Brain Tumor Using Tsallis Entropy and Regularised Level Set. Proceedings of 2nd International Conference on Micro-Electronics, Electromagnetics and Telecommunications.

[59] Cabria I., Gondra I. Automated Localization of Brain Tumors in MRI Using Potential-K-Means Clustering Algorithm. Proceedings of the 2015 12th Conference on Computer and Robot Vision.

[60] Suraj N.S.S.K., Muppalla V., Sanghani P., Ren H. Comparative Study of Unsupervised Segmentation Algorithms for Delineating Glioblastoma Multiforme Tumour. Proceedings of the 2018 3rd International Conference on Advanced Robotics and Mechatronics (ICARM).

[61] Mehidi I., Belkhiat D.E.C., Jabri D. An Improved Clustering Method Based on K-Means Algorithm for MRI Brain Tumor Segmentation. Proceedings of the 2019 6th International Conference on Image and Signal Processing and their Applications (ISPA).

[62] Rundo L., Militello C., Tangherloni A., Russo G., Vitabile S., Gilardi M.C., Mauri G. (2017). NeXt for neuro-radiosurgery: A fully automatic approach for necrosis extraction in brain tumor MRI using an unsupervised machine learning technique. Int. J. Imaging Syst. Technol..

[63] Chandra G.R., Rao K.R.H. (2016). Tumor Detection In Brain Using Genetic Algorithm. Procedia Comput. Sci..

[64] Rundo L., Militello C., Russo G., Vitabile S., Gilardi M.C., Mauri G. (2017). GTVcut for neuro-radiosurgery treatment planning: An MRI brain cancer seeded image segmentation method based on a cellular automata model. Nat. Comput..

[65] Ayachi R., Ben Amor N., Sossai C., Chemello G. (2009). Brain Tumor Segmentation Using Support Vector Machines. Symbolic and Quantitative Approaches to Reasoning with Uncertainty.

[66] Cui B., Xie M., Wang C. A Deep Convolutional Neural Network Learning Transfer to SVM-Based Segmentation Method for Brain Tumor. Proceedings of the 2019 IEEE 11th International Conference on Advanced Infocomm Technology (ICAIT).

[67] Zhang N., Ruan S., Lebonvallet S., Liao Q., Zhu Y. Multi-kernel SVM based classification for brain tumor segmentation of MRI multi-sequence. Proceedings of the 2009 16th IEEE International Conference on Image Processing (ICIP).

[68] Chen W., Qiao X., Liu B., Qi X., Wang R., Wang X. Automatic brain tumor segmentation based on features of separated local square. Proceedings of the 2017 Chinese Automation Congress (CAC).

[69] Chithambaram T., Perumal K. Brain tumor segmentation using genetic algorithm and ANN techniques. Proceedings of the 2017 IEEE International Conference on Power, Control, Signals and Instrumentation Engineering (ICPCSI).

[70] Bougacha A., Boughariou J., Slima M.B., Hamida A.B., Mahfoudh K.B., Kammoun O., Mhiri C. Comparative study of supervised and unsupervised classification methods: Application to automatic MRI glioma brain tumors segmentation. Proceedings of the 2018 4th International Conference on Advanced Technologies for Signal and Image Processing (ATSIP).

[71] Ma C., Luo G., Wang K. (2018). Concatenated and Connected Random Forests With Multiscale Patch Driven Active Contour Model for Automated Brain Tumor Segmentation of MR Images. IEEE Trans. Med. Imaging.

[72] Tang H., Lu H., Liu W., Tao X. Tumor segmentation from single contrast MR images of human brain. Proceedings of the 2015 IEEE 12th International Symposium on Biomedical Imaging (ISBI).

[73] Csaholczi S., Kovacs L., Szilagyi L. Automatic Segmentation of Brain Tumor Parts from MRI Data Using a Random Forest Classifier. Proceedings of the 2021 IEEE 19th World Symposium on Applied Machine Intelligence and Informatics (SAMI).

[74] Pinto A., Pereira S., Dinis H., Silva C.A., Rasteiro D.M.L.D. Random decision forests for automatic brain tumor segmentation on multi-modal MRI images. Proceedings of the 2015 IEEE 4th Portuguese Meeting on Bioengineering (ENBENG).

[75] Hatami T., Hamghalam M., Reyhani-Galangashi O., Mirzakuchaki S. A Machine Learning Approach to Brain Tumors Segmentation Using Adaptive Random Forest Algorithm. Proceedings of the 2019 5th Conference on Knowledge Based Engineering and Innovation (KBEI).

[76] Fulop T., Gyorfi A., Csaholczi S., Kovacs L., Szilagyi L. Brain Tumor Segmentation from Multi-Spectral MRI Data Using Cascaded Ensemble Learning. Proceedings of the 2020 IEEE 15th International Conference of System of Systems Engineering (SoSE).

[77] Bakas S., Akbari H., Sotiras A., Bilello M., Rozycki M., Kirby J.S., Freymann J.B., Farahani K., Davatzikos C. (2017). Advancing The Cancer Genome Atlas glioma MRI collections with expert segmentation labels and radiomic features. Sci. Data.

[78] Bakas S., Akbari H., Sotiras A., Bilello M., Rozycki M., Kirby J., Freymann J., Farahani K., Davatzikos C. (2017). Segmentation Labels for the Pre-operative Scans of the TCGA-GBM collection. Cancer Imaging Arch..

[79] Tobon-Gomez C., Geers A.J., Peters J., Weese J., Pinto K., Karim R., Ammar M., Daoudi A., Margeta J., Sandoval Z. (2015). Benchmark for Algorithms Segmenting the Left Atrium From 3D CT and MRI Datasets. IEEE Trans. Med Imaging.

[80] Menze B.H., Jakab A., Bauer S., Kalpathy-Cramer J., Farahani K., Kirby J., Burren Y., Porz N., Slotboom J., Wiest R. (2015). The Multimodal Brain Tumor Image Segmentation Benchmark (BRATS). IEEE Trans. Med Imaging.

[81] Pereira S., Pinto A., Alves V., Silva C.A. (2016). Brain Tumor Segmentation Using Convolutional Neural Networks in MRI Images. IEEE Trans. Med. Imaging.

[82] Deng W., Shi Q., Wang M., Zheng B., Ning N. (2020). Deep Learning-Based HCNN and CRF-RRNN Model for Brain Tumor Segmentation. IEEE Access.

[83] Ding Y., Li C., Yang Q., Qin Z., Qin Z. (2019). How to Improve the Deep Residual Network to Segment Multi-Modal Brain Tumor Images. IEEE Access.

[84] Ding Y., Chen F., Zhao Y., Wu Z., Zhang C., Wu D. (2019). A Stacked Multi-Connection Simple Reducing Net for Brain Tumor Segmentation. IEEE Access.

[85] Ali M., Gilani S.O., Waris A., Zafar K., Jamil M. (2020). Brain Tumour Image Segmentation Using Deep Networks. IEEE Access.

[86] Razzak M.I., Imran M., Xu G. (2019). Efficient Brain Tumor Segmentation With Multiscale Two-Pathway-Group Conventional Neural Networks. IEEE J. Biomed. Health Inform..

[87] Aboelenein N.M., Songhao P., Koubaa A., Noor A., Afifi A. (2020). HTTU-Net: Hybrid Two Track U-Net for Automatic Brain Tumor Segmentation. IEEE Access.

[88] Wang G., Li W., Zuluaga M.A., Pratt R., Patel P.A., Aertsen M., Doel T., David A.L., Deprest J., Ourselin S. (2018). Interactive Medical Image Segmentation Using Deep Learning With Image-Specific Fine Tuning. IEEE Trans. Med. Imaging.

[89] Hao J., Li X., Hou Y. (2020). Magnetic Resonance Image Segmentation Based on Multi-Scale Convolutional Neural Network. IEEE Access.

[90] Zhou T., Canu S., Ruan S. (2020). Fusion based on attention mechanism and context constraint for multi-modal brain tumor segmentation. Comput. Med Imaging Graph..

[91] Ye F., Zheng Y., Ye H., Han X., Li Y., Wang J., Pu J. (2021). Parallel pathway dense neural network with weighted fusion structure for brain tumor segmentation. Neurocomputing.

[92] Sun J., Peng Y., Guo Y., Li D. (2021). Segmentation of the multimodal brain tumor image used the multi-pathway architecture method based on 3D FCN. Neurocomputing.

[93] Ben naceur M., Akil M., Saouli R., Kachouri R. (2020). Fully automatic brain tumor segmentation with deep learning-based selective attention using overlapping patches and multi-class weighted cross-entropy. Med. Image Anal..

[94] Naser M.A., Deen M.J. (2020). Brain tumor segmentation and grading of lower-grade glioma using deep learning in MRI images. Comput. Biol. Med..

[95] Zhou Z., He Z., Jia Y. (2020). AFPNet: A 3D fully convolutional neural network with atrous-convolution feature pyramid for brain tumor segmentation via MRI images. Neurocomputing.

[96] Li H., Li A., Wang M. (2019). A novel end-to-end brain tumor segmentation method using improved fully convolutional networks. Comput. Biol. Med..

[97] Zhang J., Zeng J., Qin P., Zhao L. (2021). Brain tumor segmentation of multi-modality MR images via triple intersecting U-Nets. Neurocomputing.

[98] Xu F., Ma H., Sun J., Wu R., Liu X., Kong Y. LSTM Multi-modal UNet for Brain Tumor Segmentation. Proceedings of the 2019 IEEE 4th International Conference on Image, Vision and Computing (ICIVC).

[99] Kleihues P., Louis D.N., Scheithauer B.W., Rorke L.B., Reifenberger G., Burger P.C., Cavenee W.K. (2002). The WHO Classification of Tumors of the Nervous System. J. Neuropathol. Exp. Neurol..

[100] Badža M.M., Barjaktarović M.Č. (2020). Classification of Brain Tumors from MRI Images Using a Convolutional Neural Network. Appl. Sci..

[101] Tandel G.S., Biswas M., Kakde O.G., Tiwari A., Suri H.S., Turk M., Laird J., Asare C., Ankrah A.A., Khanna N.N. (2019). A Review on a Deep Learning Perspective in Brain Cancer Classification. Cancers.

[102] Quon J., Bala W., Chen L., Wright J., Kim L., Han M., Shpanskaya K., Lee E., Tong E., Iv M. (2020). Deep Learning for Pediatric Posterior Fossa Tumor Detection and Classification: A Multi-Institutional Study. Am. J. Neuroradiol..

[103] Díaz-Pernas F.J., Martínez-Zarzuela M., Antón-Rodríguez M., González-Ortega D. (2021). A Deep Learning Approach for Brain Tumor Classification and Segmentation Using a Multiscale Convolutional Neural Network. Healthcare.

[104] Deepak S., Ameer P. (2019). Brain tumor classification using deep CNN features via transfer learning. Comput. Biol. Med..

[105] Paul J.S., Plassard A.J., Landman B.A., Fabbri D., Krol A., Gimi B. (2017). Deep learning for brain tumor classification. Medical Imaging 2017: Biomedical Applications in Molecular, Structural, and Functional Imaging.

[106] Khan H.A., Jue W., Mushtaq M., Mushtaq M.U. (2020). Brain tumor classification in MRI image using convolutional neural network. Math. Biosci. Eng..

[107] Dangeti P. (2017). Statistics for Machine Learning.

[108] Ahmmed R., Swakshar A.S., Hossain M.F., Rafiq M.A. Classification of tumors and it stages in brain MRI using support vector machine and artificial neural network. Proceedings of the 2017 International Conference on Electrical, Computer and Communication Engineering (ECCE).

[109] Ismael M.R., Abdel-Qader I. Brain Tumor Classification via Statistical Features and Back-Propagation Neural Network. Proceedings of the 2018 IEEE International Conference on Electro/Information Technology (EIT).

[110] Sathi K.A., Islam M.S. Hybrid Feature Extraction Based Brain Tumor Classification using an Artificial Neural Network. Proceedings of the 2020 IEEE 5th International Conference on Computing Communication and Automation (ICCCA).

[111] Shree N.V., Kumar T.N.R. (2018). Identification and classification of brain tumor MRI images with feature extraction using DWT and probabilistic neural network. Brain Inform..

[112] Ramdlon R.H., Kusumaningtyas E.M., Karlita T. Brain Tumor Classification Using MRI Images with K-Nearest Neighbor Method. Proceedings of the 2019 International Electronics Symposium (IES).

[113] Garg G., Garg R. (2021). Brain Tumor Detection and Classification based on Hybrid Ensemble Classifier. arXiv.

[114] Engy N., Salam N.M., Al-Atabany W. (2018). Evaluating the Efficiency of different Feature Sets on Brain Tumor Classification in MR Images. Int. J. Comput. Appl..

[115] Gurbina M., Lascu M., Lascu D. Tumor Detection and Classification of MRI Brain Image using Different Wavelet Transforms and Support Vector Machines. Proceedings of the 2019 42nd International Conference on Telecommunications and Signal Processing (TSP).

[116] Ali H.M. (2018). MRI Medical Image Denoising by Fundamental Filters. High-Resolution Neuroimaging—Basic Physical Principles and Clinical Applications.

[117] Liu L., Yang H., Fan J., Liu R.W., Duan Y. (2019). Rician noise and intensity nonuniformity correction (NNC) model for MRI data. Biomed. Signal Process. Control.

[118] Ramesh S., Sasikala S., Paramanandham N. (2021). Segmentation and classification of brain tumors using modified median noise filter and deep learning approaches. Multimed. Tools Appl..

[119] Ravikumar Gurusamy D.V.S. (2017). A Machine Learning Approach for MRI Brain Tumor Classification. Comput. Mater. Contin..

[120] Li M., Wang H., Shang Z., Yang Z., Zhang Y., Wan H. (2020). Ependymoma and pilocytic astrocytoma: Differentiation using radiomics approach based on machine learning. J. Clin. Neurosci..

[121] Kaplan K., Kaya Y., Kuncan M., Ertunç H.M. (2020). Brain tumor classification using modified local binary patterns (LBP) feature extraction methods. Med. Hypotheses.

[122] Kang J., Ullah Z., Gwak J. (2021). MRI-Based Brain Tumor Classification Using Ensemble of Deep Features and Machine Learning Classifiers. Sensors.

[123] Amin J., Sharif M., Raza M., Saba T., Rehman A. Brain Tumor Classification: Feature Fusion. Proceedings of the 2019 International Conference on Computer and Information Sciences (ICCIS).

[124] Baranwal S.K., Jaiswal K., Vaibhav K., Kumar A., Srikantaswamy R. Performance analysis of Brain Tumour Image Classification using CNN and SVM. Proceedings of the 2020 Second International Conference on Inventive Research in Computing Applications (ICIRCA).

[125] Gumaei A., Hassan M.M., Hassan M.R., Alelaiwi A., Fortino G. (2019). A Hybrid Feature Extraction Method With Regularized Extreme Learning Machine for Brain Tumor Classification. IEEE Access.

[126] Minz A., Mahobiya C. MR Image Classification Using Adaboost for Brain Tumor Type. Proceedings of the 2017 IEEE 7th International Advance Computing Conference (IACC).

[127] Gayathri S., Wise D.J.W., Janani V., Eleaswari M., Hema S. Analyzing, Detecting and Automatic Classification of Different Stages of Brain Tumor Using Region Segmentation and Support Vector Machine. Proceedings of the 2020 International Conference on Electronics and Sustainable Communication Systems (ICESC).

[128] Sarkar A., Maniruzzaman M., Ahsan M.S., Ahmad M., Kadir M.I., Islam S.M.T. Identification and Classification of Brain Tumor from MRI with Feature Extraction by Support Vector Machine. Proceedings of the 2020 International Conference for Emerging Technology (INCET).

[129] Mathew A.R., Anto P.B. Tumor detection and classification of MRI brain image using wavelet transform and SVM. Proceedings of the 2017 International Conference on Signal Processing and Communication (ICSPC).

[130] Cinarer G., Emiroglu B.G. Classificatin of Brain Tumors by Machine Learning Algorithms. Proceedings of the 2019 3rd International Symposium on Multidisciplinary Studies and Innovative Technologies (ISMSIT).

[131] Lavanyadevi R., Machakowsalya M., Nivethitha J., Kumar A.N. Brain tumor classification and segmentation in MRI images using PNN. Proceedings of the 2017 IEEE International Conference on Electrical, Instrumentation and Communication Engineering (ICEICE).

[132] Amin J., Sharif M., Yasmin M., Fernandes S.L. (2020). A distinctive approach in brain tumor detection and classification using MRI. Pattern Recognit. Lett..

[133] Prabha S., Raghav R., Moulya C., Preethi K.G., Sankaran K. Fusion based Brain Tumor Classification using Multiscale Transform Methods. Proceedings of the 2020 International Conference on Communication and Signal Processing (ICCSP).

[134] Wasule V., Sonar P. Classification of brain MRI using SVM and KNN classifier. Proceedings of the 2017 Third International Conference on Sensing, Signal Processing and Security (ICSSS).

[135] Sachdeva J., Kumar V., Gupta I., Khandelwal N., Ahuja C.K. (2016). A package-SFERCB-“Segmentation, feature extraction, reduction and classification analysis by both SVM and ANN for brain tumors”. Appl. Soft Comput..

[136] Keerthana K., Xavier S. An Intelligent System for Early Assessment and Classification of Brain Tumor. Proceedings of the 2018 Second International Conference on Inventive Communication and Computational Technologies (ICICCT).

[137] Yin B., Wang C., Abza F. (2020). New brain tumor classification method based on an improved version of whale optimization algorithm. Biomed. Signal Process. Control.

[138] Cheng J. (2017). Brain Tumor Dataset. https://figshare.com/articles/dataset/brain_tumor_dataset/1512427.

[139] Gaikwad S.B., Joshi M.S. (2015). Brain Tumor Classification using Principal Component Analysis and Probabilistic Neural Network. Int. J. Comput. Appl..

[140] Kumar A., Ashok A., Ansari M.A. Brain Tumor Classification Using Hybrid Model Of PSO And SVM Classifier. Proceedings of the 2018 International Conference on Advances in Computing, Communication Control and Networking (ICACCCN).

[141] Ge C., Gu I.Y.H., Jakola A.S., Yang J. (2020). Enlarged Training Dataset by Pairwise GANs for Molecular-Based Brain Tumor Classification. IEEE Access.

[142] Bakas S., Akbari H., Sotiras A., Bilello M., Rozycki M., Kirby J., Freymann J., Farahani K., Davatzikos C. (2017). Segmentation Labels for the Pre-operative Scans of the TCGA-LGG collection. Cancer Imaging Arch..

[143] Sultan H.H., Salem N.M., Al-Atabany W. (2019). Multi-Classification of Brain Tumor Images Using Deep Neural Network. IEEE Access.

[144] Scarpace L., Flanders A.E., Jain R., Mikkelsen T., Andrews D.W. (2019). Data from Rembrandt. https://wiki.cancerimagingarchive.net/display/Public/REMBRANDT.

[145] Huang Z., Du X., Chen L., Li Y., Liu M., Chou Y., Jin L. (2020). Convolutional Neural Network Based on Complex Networks for Brain Tumor Image Classification With a Modified Activation Function. IEEE Access.

[146] Afshar P., Mohammadi A., Plataniotis K.N. (2020). BayesCap: A Bayesian Approach to Brain Tumor Classification Using Capsule Networks. IEEE Signal Process. Lett..

[147] Ucuzal H., YASAR S., Colak C. Classification of brain tumor types by deep learning with convolutional neural network on magnetic resonance images using a developed web-based interface. Proceedings of the 2019 3rd International Symposium on Multidisciplinary Studies and Innovative Technologies (ISMSIT).

[148] Noreen N., Palaniappan S., Qayyum A., Ahmad I., Imran M., Shoaib M. (2020). A Deep Learning Model Based on Concatenation Approach for the Diagnosis of Brain Tumor. IEEE Access.

[149] Rehman A., Naz S., Razzak M.I., Akram F., Imran M. (2019). A Deep Learning-Based Framework for Automatic Brain Tumors Classification Using Transfer Learning. Circuits Syst. Signal Process..

[150] Cheng Y., Qin G., Zhao R., Liang Y., Sun M. (2019). ConvCaps: Multi-input Capsule Network for Brain Tumor Classification. Neural Information Processing.

[151] Kurup R.V., Sowmya V., Soman K.P. (2019). Effect of Data Pre-processing on Brain Tumor Classification Using Capsulenet. ICICCT 2019 – System Reliability, Quality Control, Safety, Maintenance and Management.

[152] Liu D., Liu Y., Dong L. (2019). G-ResNet: Improved ResNet for Brain Tumor Classification. Neural Information Processing.

[153] Kokkalla S., Kakarla J., Venkateswarlu I.B., Singh M. (2021). Three-class brain tumor classification using deep dense inception residual network. Soft Comput..

[154] Çinarer G., Emiroğlu B.G., Yurttakal A.H. (2020). Prediction of Glioma Grades Using Deep Learning with Wavelet Radiomic Features. Appl. Sci..

[155] Erickson B., Akkus Z., Sedlar J., Korfiatis P. (2017). Data from LGG-1p19qDeletion. https://wiki.cancerimagingarchive.net/display/Public/LGG-1p19qDeletion.

[156] Abiwinanda N., Hanif M., Hesaputra S.T., Handayani A., Mengko T.R. (2018). Brain Tumor Classification Using Convolutional Neural Network. IFMBE Proceedings.

[157] Sharif M.I., Khan M.A., Alhussein M., Aurangzeb K., Raza M. (2021). A decision support system for multimodal brain tumor classification using deep learning. Complex Intell. Syst..

[158] Irmak E. (2021). Multi-Classification of Brain Tumor MRI Images Using Deep Convolutional Neural Network with Fully Optimized Framework. Iran. J. Sci. Technol. Trans. Electr. Eng..

[159] Clark K., Vendt B., Smith K., Freymann J., Kirby J., Koppel P., Moore S., Phillips S., Maffitt D., Pringle M. (2013). The Cancer Imaging Archive (TCIA): Maintaining and Operating a Public Information Repository. J. Digit. Imaging.

[160] Pei L., Vidyaratne L., Rahman M.M., Iftekharuddin K.M. (2020). Context aware deep learning for brain tumor segmentation, subtype classification, and survival prediction using radiology images. Sci. Rep..

[161] Kumar S.N., Fred A.L., Varghese P.S. (2018). An Overview of Segmentation Algorithms for the Analysis of Anomalies on Medical Images. J. Intell. Syst..

[162] Biratu E.S., Schwenker F., Debelee T.G., Kebede S.R., Negera W.G., Molla H.T. (2021). Enhanced Region Growing for Brain Tumor MR Image Segmentation. J. Imaging.

[163] Miotto R., Wang F., Wang S., Jiang X., Dudley J.T. (2017). Deep learning for healthcare: Review, opportunities and challenges. Briefings Bioinform..

[164] Zhu G., Jiang B., Tong L., Xie Y., Zaharchuk G., Wintermark M. (2019). Applications of Deep Learning to Neuro-Imaging Techniques. Front. Neurol..

[165] Sert E., Özyurt F., Doğantekin A. (2019). A new approach for brain tumor diagnosis system: Single image super resolution based maximum fuzzy entropy segmentation and convolutional neural network. Med. Hypotheses.

[166] Natekar P., Kori A., Krishnamurthi G. (2020). Demystifying Brain Tumor Segmentation Networks: Interpretability and Uncertainty Analysis. Front. Comput. Neurosci..

[167] Saleem H., Shahid A.R., Raza B. (2021). Visual interpretability in 3D brain tumor segmentation network. Comput. Biol. Med..

[168] Zeng Y., Zhang B., Zhao W., Xiao S., Zhang G., Ren H., Zhao W., Peng Y., Xiao Y., Lu Y. (2020). Magnetic Resonance Image Denoising Algorithm Based on Cartoon, Texture, and Residual Parts. Comput. Math. Methods Med..

[169] Heo Y.C., Kim K., Lee Y. (2020). Image Denoising Using Non-Local Means (NLM) Approach in Magnetic Resonance (MR) Imaging: A Systematic Review. Appl. Sci..

[170] López M.M., Frederick J.M., Ventura J. (2021). Evaluation of MRI Denoising Methods Using Unsupervised Learning. Front. Artif. Intell..

[171] Kidoh M., Shinoda K., Kitajima M., Isogawa K., Nambu M., Uetani H., Morita K., Nakaura T., Tateishi M., Yamashita Y. (2020). Deep Learning Based Noise Reduction for Brain MR Imaging: Tests on Phantoms and Healthy Volunteers. Magn. Reson. Med Sci..

[172] Higaki T., Nakamura Y., Tatsugami F., Nakaura T., Awai K. (2018). Improvement of image quality at CT and MRI using deep learning. Jpn. J. Radiol..

[173] Kim K.H., Do W.J., Park S.H. (2018). Improving resolution of MR images with an adversarial network incorporating images with different contrast. Med. Phys..

